# Cytopathological Outcomes of Knocking down Expression of Mitochondrial Complex II Subunits in *Dictyostelium discoideum*

**DOI:** 10.3390/ijms23095039

**Published:** 2022-05-01

**Authors:** Sui Lay, Xavier Pearce, Oana Sanislav, Paul Robert Fisher, Sarah Jane Annesley

**Affiliations:** Department of Physiology, Anatomy and Microbiology, La Trobe University, Bundoora, VIC 3086, Australia; s.lay@latrobe.edu.au (S.L.); x.pearce@latrobe.edu.au (X.P.); o.sanislav@latrobe.edu.au (O.S.); p.fisher@latrobe.edu.au (P.R.F.)

**Keywords:** *Dictyostelium discoideum*, Complex II, succinate dehydrogenase, mitochondria

## Abstract

Mitochondrial Complex II is composed of four core subunits and mutations to any of the subunits result in lowered Complex II activity. Surprisingly, although mutations in any of the subunits can yield similar clinical outcomes, there are distinct differences in the patterns of clinical disease most commonly associated with mutations in different subunits. Thus, mutations to the SdhA subunit most often result in mitochondrial disease phenotypes, whilst mutations to the other subunits SdhB-D more commonly result in tumour formation. The reason the clinical outcomes are so different is unknown. Here, we individually antisense-inhibited three of the Complex II subunits, SdhA, SdhB or SdhC, in the simple model organism *Dictyostelium discoideum*. Whilst SdhB and SdhC knockdown resulted in growth defects on bacterial lawns, antisense inhibition of SdhA expression resulted in a different pattern of phenotypic defects, including impairments of growth in liquid medium, enhanced intracellular proliferation of the bacterial pathogen *Legionella pneumophila* and phagocytosis. Knockdown of the individual subunits also produced different abnormalities in mitochondrial function with only SdhA knockdown resulting in broad mitochondrial dysfunction. Furthermore, these defects were shown to be mediated by the chronic activation of the cellular energy sensor AMP-activated protein kinase. Our results are in agreement with a role for loss of function of SdhA but not the other Complex II subunits in impairing mitochondrial oxidative phosphorylation and they suggest a role for AMP-activated protein kinase in mediating the cytopathological outcomes.

## 1. Introduction

Mitochondrial diseases are complex diseases resulting from mutations affecting the levels or activities of mitochondrial proteins and thereby impair the key functions of mitochondria in cells, most notably the provision of ATP by oxidative phosphorylation. Mutational reduction or loss of these essential biochemical functions of mitochondria cause mitochondrial disease, a complex array of diseases that can and do affect multiple cell types, tissues and organ systems (Schlieben et al., 2020). Nonetheless, the high energy demands of the neuromuscular and central nervous systems mean that mitochondrial disease often produces muscular and neurological deficits, including neurodegeneration. In this paper we focus on the cytopathological consequences of knocking down the expression of one particular mitochondrial protein complex, respiratory complex II or succinate dehydrogenase (Sdh). This is of particular interest because, as described below, the patterns of disease caused by mutations in different Sdh subunits are surprisingly very different and the reasons are not understood.

Sdh is a heterotetramer composed of four subunits named SdhA-D. The subunits are encoded on different chromosomes, sdhA on chromosome 5, sdhB and sdhC on chromosome 1 and sdhD on chromosome 11 [1]. It has the smallest number of subunits of all the respiratory complexes and is unique in that all of the subunits are nuclear-encoded. It is also the only complex which does not pump protons across the inner mitochondrial membrane. Another unique property of Complex II is that it participates not only in the oxidative phosphorylation pathway but also in the TCA cycle and thus has dual functions in carbon metabolism and mitochondrial respiration [2].

The four core subunits of Complex II are assembled with the assistance of four assembly factors and cofactors. The catalytic subunit, SdhA, is imported into the mitochondrial matrix and folded before FAD is covalently attached. Likewise, SdhB is also matured in the matrix by the addition of three Fe-S clusters. The flavinylated and mature SdhA subunit then forms a subcomplex by binding to the mature SdhB subunit. The SdhA/B subcomplex then binds irreversibly to the SdhC/D subunits which reside in the IMM to form the holo-enzyme [3]. SdhA is responsible for oxidising succinate to fumarate in the TCA cycle and passes the electrons to the Fe-S clusters in subunit B. SdhC and D form the membrane-bound components and together with subunit B they form a ubiquinone binding site. Electrons produced from the oxidation of succinate are then transferred to ubiquinone residing in the ubiquinone binding site and used to catalyse the reduction of ubiquinone to ubiquinol. The electrons are subsequently passed to Complex III and then Complex IV, thus contributing to oxidative phosphorylation [3].

Complex II is thus an essential component in energy production and mutations which affect its function are rare [4]. Interestingly mutations in different subunits result in very different patterns of clinical presentation and disease which can be grouped into two main categories: cancer or mitochondrial Complex II deficiency (presenting as neurodegeneration or cardiomyopathy). Mutations affecting SdhA most commonly result in a mitochondrial respiratory defect, often presenting as a progressive neurodegenerative disorder called Leigh syndrome [5] although cardiomyopathy or other neurodegenerative disorders have also been reported including late-onset optic atrophy, ataxia and leukodystrophy [6,7]. In a recent, comprehensive review of the literature, Fullerton et al. (2020) found that the vast majority of isolated Complex II deficiencies with mitochondrial phenotypes involved either homozygous or complex heterozygous alleles of sdhA. Only 5 autosomal recessive variants of sdhB and 2 variants of sdhD were found associated with mitochondrial Complex II deficiency. Mutations in sdhA are also sometimes associated with cancers, notably gastrointestinal stromal tumors (Nazar et al., 2019; McFarlane et al., 2020), about 7.5% of which involve complex II deficiency, mostly involving sdhA alleles (Rasheed and Tarjan, 2018).

In contrast with SdhA, mutations in the other subunits SdhB-D are generally associated with cancers, especially paragangliomas (PGL) and phaeochromocytomas (PCC), but also with other cancers including renal carcinoma, thyroid cancer, ovarian cancer, neuroblastoma and gastrointestinal stromal tumor [8]. There have been a small number of reports, restricted to only a few patients, of mutations to SdhB and SdhD that are associated with leukodystrophy (Fullerton et al., 2020), but generally mutations to subunits B, C and D result in neoplasty. Mutations in all subunits result in impaired Sdh activity, so it is not clear why there is such a distinction in the pattern of clinical phenotypes from the different subunit mutations.

Complex II has been well conserved throughout evolution and simple systems have been used to study its structure and function. Its crystal structure was solved in *E.coli* [9]; its assembly was aided by studies in yeast [2] and functional studies have been done in animal models [10,11].

*Dictyostelium discoideum* is a simple eukaryotic model organism which belongs to the Amoebozoa, a sister lineage to the animals and fungi that diverged from them after the plant kingdom but before the separation of the animal and fungal kingdoms [12]. The *D. discoideum* genome encodes subunits of all five mitochondrial respiratory complexes including Complex I, in contrast to some fungi, including *Saccharomyces cerevisae*. Yet, unlike animals but like most other eukaryotic lineages, it also contains an additional enzyme of oxidative phosphorylation called Alternative Oxidase (AOX). AOX is generally associated with maintaining respiration when the organisms are exposed to various stress conditions and can transfer electrons from Complex I or II via ubiquinone to oxygen.

The *D. discoideum* nuclear and mitochondrial genomes have been fully sequenced [13,14]. As in humans, the nuclear genome encodes homologues of all four Sdh subunits and several Complex II assembly factors. The organism is amenable to genetic manipulation, and mitochondrial disease has been created in this organism via gene knockouts of nuclear and mitochondrially encoded genes, knockdown of expression of mRNA transcripts encoding mitochondrial chaperonin 60 and treatment with ethidium bromide [15,16]. Analysis of the effects of gene mutations is facilitated in *D.discoideum* by its unique life cycle featuring unicellular and multicellular stages which provide a wide variety of phenotypes to study as readouts of the underlying signalling pathways. The growth of the unicellular amoebae is halted upon removal of nutrients which initiates a starvation-induced cascade of differentiation events beginning with the acquisition of the ability to secrete and respond chemotactically to cAMP. This results in the aggregation of approximately 100,000 cells which then proceed to form a sequence of multicellular structures including a motile slug and ultimately a fruiting body with a long slender stalk supporting a sorus, which is a droplet of spores. In *D. discoideum*, impairing mitochondrial respiration by manipulating genes encoding mitochondrial proteins results in the chronic activation of the energy-sensing protein AMP-activated protein kinase (AMPK) [17]. This chronic activation of AMPK results in a clear set of defective phenotypes namely defective slug phototaxis, altered fruiting body morphology and decreased growth rates independent of endocytosis.

AMPK is a highly conserved protein kinase that is ubiquitous amongst eukaryotic organisms. It can very sensitively detect energy stress, as it detects altered levels of AMP which rise when ATP levels drop even by a modest amount. AMPK is composed of three subunits—a catalytic α subunit, a scaffolding β subunit and a γ regulatory subunit. During energy stress AMP can bind to the γ subunit causing a conformational change of the complex, allowing phosphorylation by upstream kinases [18]. Phosphorylation and activation of AMPK can also occur independently of AMP binding in response to oxidative stress or elevated calcium levels, mediated respectively by the upstream kinases TAK1 and Ca^2+^/calmodulin-dependent protein kinase β (CaMMKβ) [19,20]. Once activated, AMPK works to restore energy levels by inhibiting energy-consuming anabolic pathways and activating energy-producing catabolic ones. One such energy-consuming process is the translation of proteins which is largely governed by a key regulator of nutrient stress called Target of Rapamycin Complex I (TORC1). Activated AMPK can phosphorylate and thereby inhibit TORC1.

Here we have used the model organism *D. discoideum* and deployed antisense inhibition to individually knockdown expression of three of the Sdh subunits (SdhA, B and C). This allowed us to investigate if the reduction or loss of function of these subunits produces different cytopathological outcomes and could potentially shed light on why mutations affecting these subunits are associated with such different clinical outcomes in humans.

## 2. Results

### 2.1. Antisense Inhibition of Expression of Sdh Subunits

**Figure 1:**
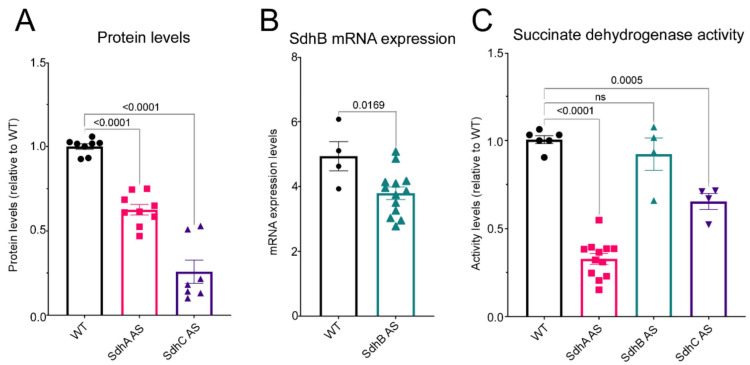
Antisense inhibition results in reduced expression and activity of Sdh. (**A**) The amount of SdhA and SdhC protein present in SdhA (SdhA AS) and SdhC (SdhC AS) antisense transformants and wild type AX2 (WT) was measured using antibodies targeted against each of the proteins. The amount of protein detected was normalised against the total protein loading and then against the wild- type protein expression. (**B**) The amount of SdhB in SdhB antisense transformants (SdhB AS) and AX2 wild type strain (WT) was determined by semiquantitative RT-PCR. Threshold values were normalised by subtraction relative to the amount of filamin mRNA. (**C**) The succinate dehydrogenase activity was measured in all the antisense inhibition transformants and normalised to the wild- type strain. SdhA and SdhC antisense transformants showed a significant reduction in Sdh activity. A small reduction in Sdh activity was also noted in the SdhB transformants; however, this was not statistically significant. In all panels the experiments were performed in triplicate within each of three independent experiments for each transformant represented by a circle (WT), square (SdhA AS), green triangle (SdhB AS) or purple triangle (SdhC AS). Error bars represent standard errors of the mean and statistical significances were determined using an ANOVA with pairwise comparisons using the least squares difference test and the Tukey method for correction of multiple comparisons. Numbers in each panel are significance probabilities in the pairwise comparisons, with nonsignificant values (adjusted *p* > 0.05) represented by ns.

To investigate the roles of three of the four subunits of Sdh in *D. discoideum* we created stable knockdown strains by transforming the *D. discoideum* parental strain AX2 with antisense inhibition constructs. Several transformants containing each construct were isolated and verified via qPCR. To determine if the expression of these antisense constructs resulted in downregulation of the protein, we performed western blots or qRT-PCR to measure the expression of each of the Sdh subunits in question. As seen in Figure 1A antisense inhibition of SdhA and SdhC resulted in a reduced amount of the corresponding protein in the transformants.

Unfortunately, we were not able to detect SdhB using commercially available antibodies and hence could not assay the amount of SdhB protein. Instead, we measured the level of SdhB mRNA using qRT-PCR and gene-specific primers which amplified a 100 bp region of the gene. Figure 1B illustrates that antisense inhibition of SdhB did result in a reduction of SdhB mRNA. We performed a succinate dehydrogenase activity assay and showed that antisense inhibition of all three of the subunits resulted in reduced Sdh activity; however, this did not reach statistical significance for SdhB transformants (Figure 1C).

### 2.2. Sdh Subunits Have Differing Roles in Growth Not Attributable to Roles in Endocytosis

**Figure 2:**
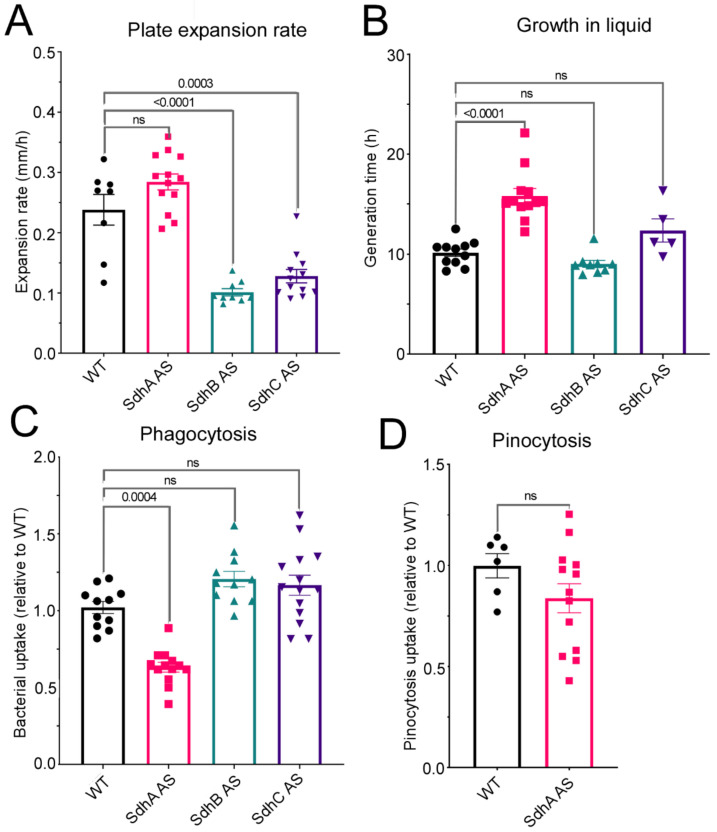
Sdh subunits play differing roles in cell growth and endocytosis. (**A**) Wild type and transformant strains were grown on lawns of *E. coli* B2 and the diameter of *D. discoideum* plaques was measured twice daily for five days and the rate of plaque expansion (mm/h) was calculated. (**B**) Cells were inoculated at an initial concentration of 1 × 10^4^ cells/mL and grown shaking at 21 °C for a period of five days. A small amount of medium was removed and counted twice daily and used to calculate the generation time. (**C**) The ability of wild type AX2 and transformants to phagocytose a fluorescently labelled bacterium (*E.coli* expressing Ds-Red) was measured and normalised to wild type. (**D**) Wild type and SdhA transformants were resuspended to a density of 1 × 10^7^ cells/mL and incubated in HL-5 media containing FITC-dextran. Uptake of FITC-dextran was measured after 70 h using a fluorometer and values were normalised against the wild type. In all cases, experiments were performed in triplicate in 3 individual experiments for each transformant and represented by a circle (WT), square (SdhA AS), green triangle (SdhB AS) or purple triangle (SdhC AS). Error bars represent standard errors of the mean and statistical significances were determined using an ANOVA with pairwise comparisons using the least-squares difference test. The Tukey method was used for correction of multiple comparisons. Numbers in each panel are significance probabilities in the pairwise comparisons with non-significant values (adjusted *p* > 0.05) represented by ns.

To elucidate the cellular roles of each of the subunits in pathways associated with either mitochondrial dysfunction or cell growth and proliferation, we first investigated the impact of their knockdown on growth and endocytosis. Altered cell proliferation plays clear roles in cancer phenotypes and previously we have reported that mitochondrial dysfunction in *D. discoideum* results in a consistent set of defective phenotypes, one of these being a defect in growth which is not due to or accompanied by a defect in endocytosis. Here we measured the growth of *D. discoideum* amoebae on both bacterial cultures grown on agar plates and also axenically in liquid HL-5 medium. SdhA antisense transformants grew slower in liquid media as evident by the longer generation times seen in Figure 2B but exhibited normal growth rates on bacterial lawns (Figure 2A). By contrast, SdhB and SdhC antisense transformants grew at the same rate as the wild- type strain in axenic media (Figure 2B) but grew slower on bacterial lawns (Figure 2A). To determine if these defects were due to a defect in endocytosis, we performed phagocytosis assays for Sdh transformants and found that the decreased plaque expansion rate of SdhB and C transformants was not due to a defect in phagocytosis. This is reminiscent of the phenotypic patterns previously shown to result from mitochondrial respiratory dysfunction, except that in that case growth in liquid was also impaired.

We also measured phagocytosis for SdhA transformants even though they did not show a defect in growth on bacterial lawns and unexpectedly observed a significant decrease in phagocytosis rates. Given this decreased phagocytosis and the observed decrease in growth in liquid, we measured the macropinocytosis rate in SdhA antisense transformants and whilst the rate was also decreased, the change did not reach statistical significance. Therefore, SdhB and C play different roles in growth than SdhA, SdhB and C are needed for growth on bacterial lawns and SdhA for growth in liquid medium. Furthermore, SdhA is also needed for phagocytosis whereas SdhB and C are not.

### 2.3. Sdh Subunits Are Not Required for Efficient Multicellular Development

**Figure 3:**
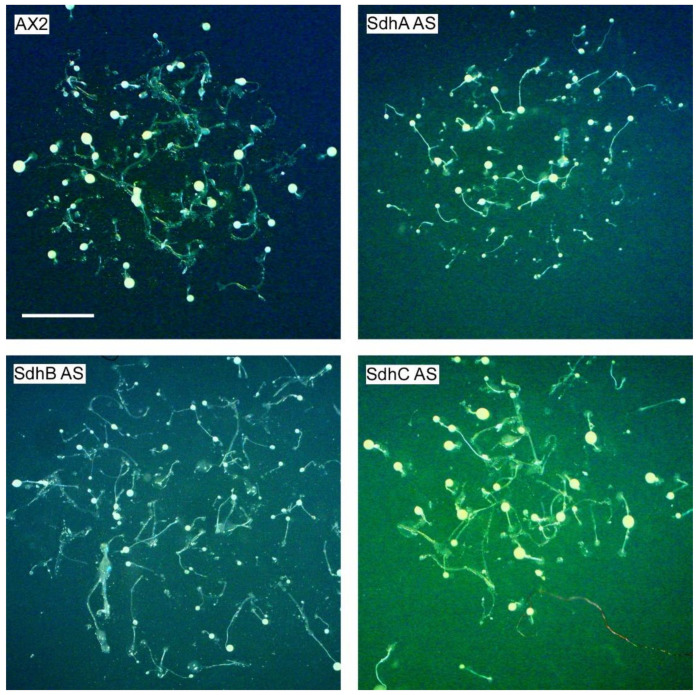
Sdh knockdown mutants have no defect in fruiting body formation. *D.discoideum* wild type (AX2) and Sdh antisense inhibited transformants were plated onto water agar and allowed to develop. Aerial images were taken, and the scale bar represents 1 mm. All Sdh strains; SdhA (SdhA AS), SdhB (SdhB AS) and SdhC (SdhC AS) antisense transformants formed fruiting bodies which resembled the wild type strain AX2.

*Dictyostelium* amoebae live in forest soil and obtain nutrients by phagocytosis of bacteria. When the food source becomes limited, the amoebae initiate a developmental cascade with approximately 100,000 amoebae aggregating chemotactically to form a mound that further develops into a standing finger that can fall over to become a migrating slug. After a variable period of migration, the slug culminates to form a fruiting body consisting of a long slender stalk sitting on a basal disc and holding up a sorus full of spores. Here we plated a scraping of amoebae grown on bacteria onto non-nutrient water agar and took aerial pictures (Figure 3). All the Sdh mutants formed fruiting bodies which resembled the wild type parental strain AX2 suggesting that none of the subunits are required for efficient multicellular development.

### 2.4. Steady-State Parameters of Mitochondrial Respiratory Function Are Affected Only in SdhA Antisense Transformants

**Figure 4:**
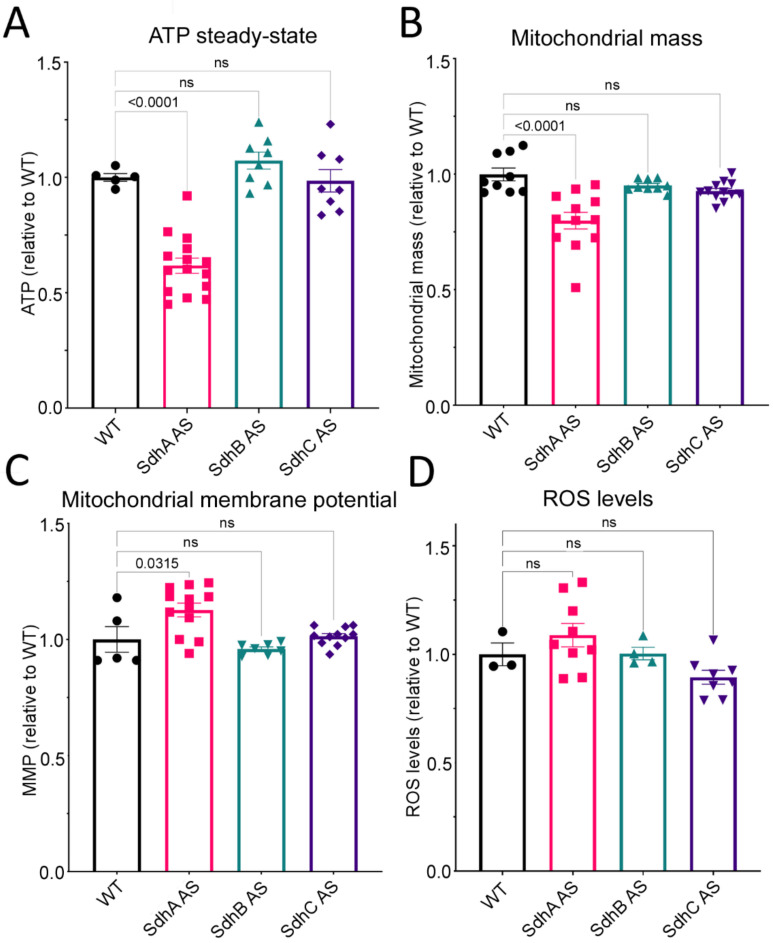
SdhA but not SdhB or C display altered mitochondrial parameters. Wild type and Sdh antisense transformants were grown axenically in HL-5 medium and the cells were harvested, then incubated with various dyes to measure ATP steady-state levels (**A**), mitochondrial mass (**B**), mitochondrial membrane potential (**C**) and reactive oxygen species (ROS) (**D**). Experiments were performed in triplicate in 3 independent experiments and error bars represent standard errors of the mean. Each transformant is represented by a circle (WT), square (SdhA AS), green triangle (SdhB AS) or purple triangle (SdhC AS). SdhA antisense transformants exhibit decreased ATP steady-state levels, decreased mitochondrial mass and increased mitochondrial membrane potential. Knockdown of any of the Sdh subunits had no effect on the levels of ROS. Error bars represent standard errors of the mean and statistical significances were determined using an ANOVA with pairwise comparisons using the least-squares difference test and the Tukey method for correction of multiple comparisons. Numbers in each panel are significance probabilities in the pairwise comparisons with non-significant values (adjusted *p* > 0.05) represented by ns.

Knockdown of SdhA-C subunits did not result in a clear set of defective phenotypes that exactly match the typical patterns for mitochondrial respiratory dysfunction, namely impaired growth in both liquid and on bacterial lawns, no defect in endocytosis and altered fruiting body morphology [17,21]. It was therefore of interest to measure directly several parameters of mitochondrial function (Figure 4).

We measured the total mitochondrial mass using a fluorescent dye (Mitotracker Green) which binds to the mitochondrial membrane regardless of the mitochondrial membrane potential. The results showed that SdhA antisense transformants exhibit a significant reduction in their mitochondrial mass compared to the wild type strain (Figure 4B). Steady-state ATP levels were also lower in the SdhA antisense transformants (Figure 4A), suggesting a decrease in mitochondrial oxidative phosphorylation to match the decreased mitochondrial mass. Consistent with these findings, the SdhA antisense transformants also exhibited an increase in mitochondrial membrane potential (MMP) compared to the wild type strain. This indicates an altered balance between the production of the proton gradient by electron transport and its reduced consumption by ATP synthesis and other mitochondrial membrane transport processes. By contrast, the knockdown of SdhB and SdhC expression had no significant effect on the ATP steady-state, mitochondrial mass or membrane potential. These results clearly implicate SdhA, but not SdhB or C in these aspects of mitochondrial function. This could be related to the fact that the SdhB and C subunits do not serve as proton pumps that contribute directly to the membrane potential, but the catalytic activity of complex II (SdhA) plays a vital role in the TCA cycle’s provision of NADH to drive oxidative phosphorylation. None of the three groups of Sdh knockdown transformants exhibited significant changes in Reactive Oxygen Species levels (Figure 4D).

### 2.5. Intracellular Legionella Proliferation Is Elevated in SdhA Antisense Transformants

**Figure 5:**
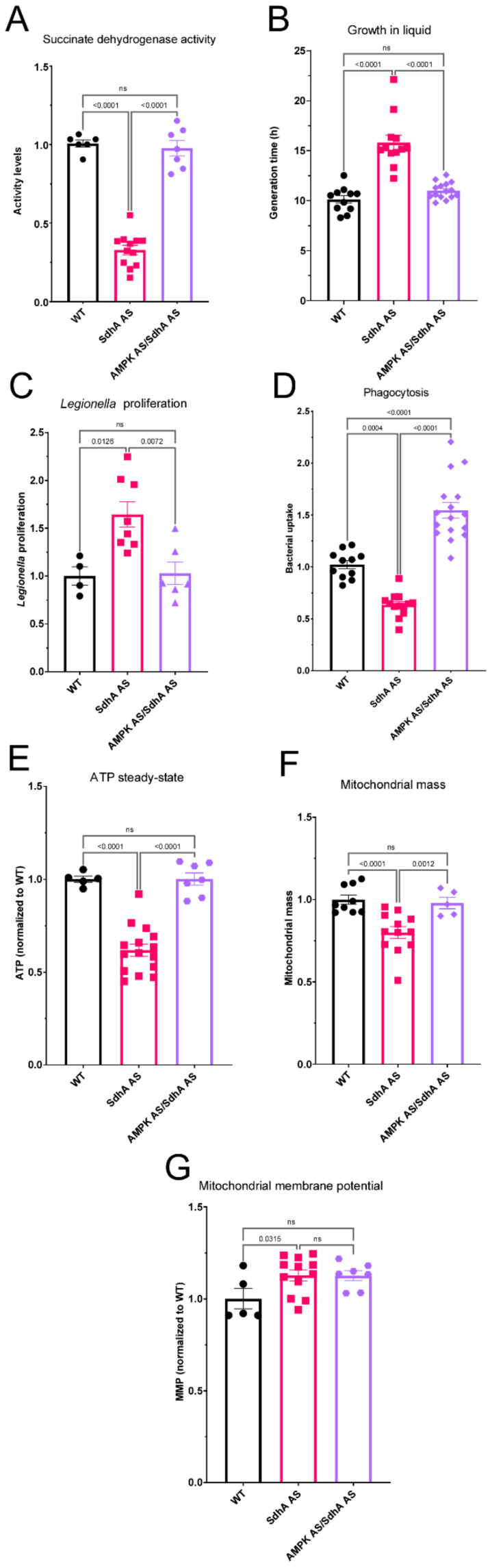
Defective SdhA phenotypes are mediated by AMPK. Wild type (Black circles), SdhA antisense transformants (red squares) and SdhA AMPK antisense cotransformants (purple circles) were analysed for their phenotypic behaviours. (**A**) Succinate dehydrogenase activity of transformants and cotransformants was measured and normalised to wild type levels. SdhA antisense inhibition reduces succinate dehydrogenase activity and knockdown of AMPK rescues this defect returning activity to wild type levels. (**B**) Cells were resuspended to a density of 1 × 10^4^ cells/mL and grown shaking at 21 °C for a period of five days. A small amount of medium was removed and counted twice daily and used to calculate the generation time. Antisense inhibition of SdhA resulted in a longer generation time and this was rescued by antisense inhibition of AMPK. (**C**) *Legionella* proliferation was measured by creating a monolayer of *D. discoideum* amoebae in a cell culture plate and infecting these with *L. pneumophila* Corby. The maximum *L. pneumophila* proliferation levels were normalised to those in the WT (AX2). Antisense inhibition of SdhA resulted in an increased *Legionella* proliferation and this was rescued by antisense inhibiting AMPK. (**D**) The ability of strains to phagocytose a fluorescently labelled bacterium (*E.coli* expressing Ds-Red) was measured and normalised to wild type. Antisense inhibition of SdhA resulted in a reduced phagocytosis rate compared to wild type and this was rescued by antisense inhibition of AMPK. (**E**) ATP steady-state levels were measured using a luminescent dye (luciferin) and values were normalised to the wild type. SdhA antisense inhibition resulted in a reduction that was rescued by antisense inhibition of AMPK. (**F**) Strains were incubated with the mitochondrial dye MitoTracker Green, and the fluorescence was measured and normalised to wild type levels. Antisense inhibition of SdhA reduced the mitochondrial mass and antisense inhibition of AMPK rescued this defect. (**G**) The mitochondrial membrane potential (MMP), as calculated by the ratio of Mitotracker Red to Mitotracker Green fluorescence, was measured and normalised to wild type levels. The MMP was significantly greater than wild type in SdhA antisense transformants, but not in the transformants in which AMPK was also antisense-inhibited. Error bars represent standard errors of the mean and statistical significances were determined using an ANOVA with pairwise comparisons using the least-squares difference test and the Tukey method for correction of multiple comparisons. Non-significant values (adjusted *p* > 0.05) are represented by ns.

Amongst the many adverse clinical outcomes common in mitochondrial disease in humans is an elevated susceptibility to infection, particularly respiratory infection. In a previous investigation of this in the *Dictyostelium* mitochondrial disease model, we found that intracellular proliferation of the bacterial intracellular pathogen *Legionella pneumophila* is elevated in mitochondrially diseased cells [22]. Since SdhA knockdown had caused an alteration of steady-state parameters of mitochondrial function, we assayed *Legionella* proliferation in our SdhA knockdown cells and found that it was enhanced, providing another phenotype in which the cytopathological outcomes of SdhA knockdown were similar to those caused by mitochondrial respiratory dysfunction in the *Dictyostelium* model (Figure 5D).

### 2.6. Defective SdhA Phenotypes Are Driven by Hyperactive AMPK

**Figure 6:**
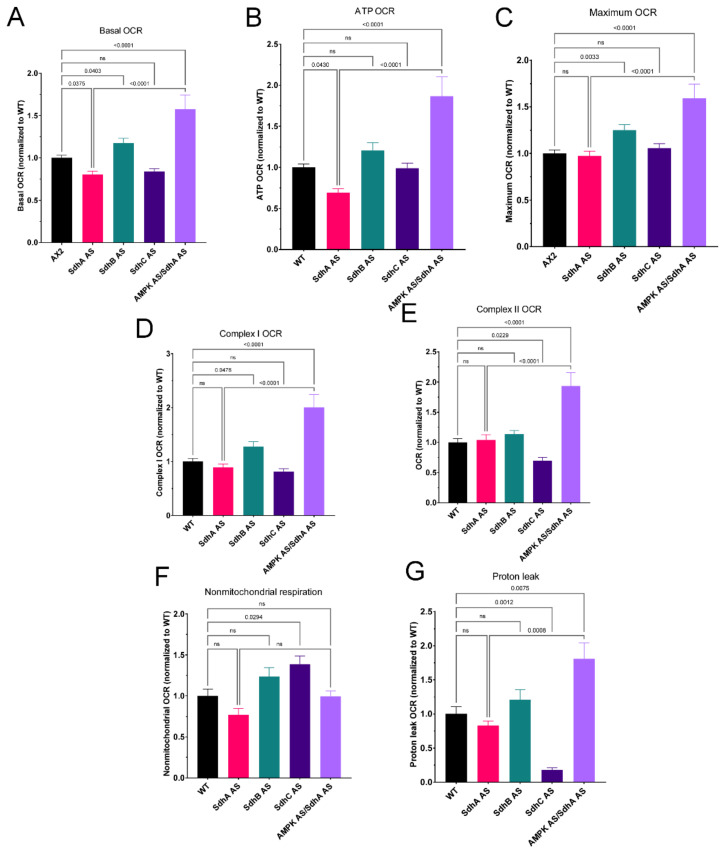
SdhA reduces ATP production, and this is rescued by AMPK knockdown. SdhB and C subunits alter different parameters of mitochondrial respiration to SdhA. In each experiment, cells of the wild type and Sdh antisense transformants or SdhA/AMPK antisense cotransformants were plated into four wells per sample of a Seahorse XFe24 plate. Mitochondrial respiration was measured by the rate of oxygen consumption. The basal respiration rate (**A**) was measured followed by the addition of DCCD (dicyclohexylcarbodimide), CCCP (carbonyl cyanide m-chlorophenyl hydrazone), and rotenone. Then either Antimycin A (complex III inhibitor) or BHAM (benzohydroxamic acid, AOX inhibitor) was added to the wells. Total activity for Complex II was calculated by adding the effects of Antimycin A and BHAM. All OCR measurements were normalised to the wild type strain in each experiment. Panels B–G represent each component of mitochondrial respiration: ATP synthesis (**B**), Maximum capacity (**C**), Complex I (**D**), Complex II total which is the combination of Complex II/III/IV and Complex II/AOX (**E**), oxygen consumption by nonmitochondrial processes (**F**) and oxygen consumption attributable to mitochondrial processes that do not generate ATP termed proton “leak” (**G**). The spare capacity is the difference between the maximum, uncoupled respiratory capacity of the cells and the basal respiration rate calculated as maximum OCR minus basal OCR.

The alterations to parameters of mitochondrial function in SdhA antisense transformants led us to investigate whether they and the other defective phenotypes were due to altered AMPK signalling. A reduction in ATP is known to activate AMPK. In addition, we have previously shown that mitochondrial dysfunction phenotypes are due to the chronic activation of the energy sensor AMP-activated protein kinase (AMPK) [17,22]. Knockdown of AMPK in mitochondrially diseased cells rescued the defects of impaired cell growth and increased *Legionella* proliferation [17,22]. Knockdown of AMPK alone has been shown previously to result in increases in axenic growth and growth on bacterial lawns without any effect on endocytosis, and no significant effect on mitochondrial mass or ATP steady-state [17]. To determine if the altered phenotypes arising from SdhA knockdown were due to increased AMPK activity, we created cotransformants which contained constructs for antisense inhibition of both the SdhA and AMPKα subunits. The copy number per genome of each construct was determined via qPCR and transformants were shown to contain SdhA antisense inhibition constructs ranging from 33–190 copies, while the AMPK antisense inhibition constructs copy numbers ranged from 36–159. Copy numbers of both are high enough to cause a reduction in expression (this work and [17]). We then assayed all the phenotypes and mitochondrial parameters in these cotransformants that we had previously shown to be altered by SdhA knockdown. We found that in all cases, AMPK knockdown resulted in a rescue of the phenotype (Figure 5 and Figure 6). This shows that, as in the case of other mitochondrial disease phenotypes in the *Dictyostelium* model, all of the SdhA phenotypes result from chronic AMPK hyperactivity.

In the case of the reduced mitochondrial mass, SdhA activity and ATP steady state levels (Figure 5) this was unexpected, as AMPK is a known activator of mitochondrial biogenesis and activity. The results suggest instead, that in the SdhA antisense transformants, AMPK hyperactivity serves to constrain another pathway that would otherwise reverse these effects. An example of such a pathway is AMPK’s inhibition of TORC1 which would otherwise translationally activate mitochondrial biogenesis and function [23]. Regardless, the phenotypes and their rescue by reducing AMPK expression are broadly consistent with the known patterns of phenotypic abnormalities in the *Dictyostelium* mitochondrial disease model. This is analogous to the situation in humans in which SdhA mutations most commonly result in clinical outcomes reminiscent of other mitochondrial diseases.

### 2.7. Reduced Expression of Different Sdh Subunits Results in Different Abnormalities in Mitochondrial Respiratory Activity

The previous results suggest that the cytopathological outcomes of SdhA knockdown may result from an AMPK-dependent mechanism that inhibits mitochondrial biogenesis and function. To measure mitochondrial function more directly, we used Seahorse respirometry in combination with a series of pharmacological agents (Figure 6). This uses the oxygen consumption rate as a readout of mitochondrial respiration in intact cells and measures the contributions of each of the complexes and other parameters to respiratory function. We had previously noted that knocking down any of the three Sdh subunits caused a reduction in Sdh activity, but this was only significant for SdhA and SdhC. We, therefore, expected that the contribution of Complex II to mitochondrial respiration would also be decreased, yet this was only evident for SdhC antisense transformants (Figure 6E).

SdhA antisense transformants displayed a reduced basal oxygen consumption rate (OCR). The basal O_2_ consumption rate is comprised of OCR attributable to ATP production, OCR by mitochondrial processes other than ATP production (proton “leak”) and OCR by nonmitochondrial oxygen-consuming processes (nonmitochondrial OCR). The SdhA antisense transformants had reduced levels of all three of these components, but this was most severe and statistically significant only for ATP synthesis (Figure 6B). Antisense inhibition of expression of the catalytic subunit of AMPK reversed this ATP synthesis defect in SdhA knockdown cells increasing ATP synthesis rates to well beyond wild type levels. It also coordinately increased the maximum respiratory capacity and the contributions to it of both Complex I and Complex II. The results in Figure 5 showed that antisense inhibition of AMPK reversed the defect in Sdh enzyme activity as measured in vitro while the respirometric results showed that it also increases Complex II activity in mitochondrial respiration by intact cells. AMPK knockdown also resulted in an increased proton leak (Figure 6G) which encompasses diverse active transport processes that are energized by the mitochondrial membrane potential. These include such processes as the import from the cytoplasm of mitochondrial proteins and oxidizable substrates for respiration. Together these results show that knocking down AMPK expression in SdhA antisense cells resulted in a coordinate increase in mitochondrial biogenesis and respiratory activity that reversed the effects of the SdhA knockdown in the same cells.

By contrast with SdhA antisense inhibition, SdhB knockdown caused an elevated basal OCR. All three components of the basal OCR were increased coordinately in the SdhB transformants, but in no case did they individually reach statistical significance, suggesting that the significant increase in the basal OCR was due to a combination of smaller elevations of all three of its components. In a similar way, the maximum respiratory capacity (Figure 6C) of SdhB transformants and its major contributors, Complex I and Complex II, were coordinately increased (Figure 6D,E), although this did not reach statistical significance in the case of Complex II. Together these results reveal that in the SdhB antisense transformants, homeostatic pathways were activated that more than compensated for the reduced sdhB mRNA expression.

SdhC transformants as mentioned earlier were the only transformants to show a reduction in Complex II activity in intact cells (Figure 6E). These transformants also showed a significant elevation of nonmitochondrial respiration (Figure 6F) and a significant reduction of the proton leak (Figure 6G). This indicates an upregulation of O_2_ consumption by some of the cellular oxygenases and hydroxylases that directly consume molecular O_2_. As a result, the maximum uncoupled respiratory activity was unchanged, despite the reduction in Complex II. ATP synthesis rates were not diminished (Figure 6B), like the SdhB but unlike the SdhA antisense transformants.

The differing patterns of functional abnormalities in mitochondrial respiratory function when the expression of different subunits is specifically knocked down are consistent with the different phenotypic outcomes observed in the previous sections. Most strikingly, only SdhA knockdown caused a significant reduction in mitochondrial ATP synthesis rates in intact cells, and only SdhA knockdown produced altered steady state measures of mitochondrial function. Moreover, all of the phenotypic and mitochondrial respiratory outcomes of knocking down SdhA were reversed by antisense inhibition of AMPK expression.

## 3. Discussion

An abiding mystery in understanding the pathological outcomes of loss of function of individual Sdh subunits has been the distinct patterns of disease outcomes associated with mutations in the different subunits. While either cancer phenotypes or typical mitochondrial disease phenotypes (neurodegeneration and/or myopathies) can be caused by loss of function mutations to any of the subunits, the relative frequencies of these outcomes differ for the different subunits (Fullerton et al., 2020). SdhA mutations mostly cause clinical outcomes reminiscent of other mitochondrial diseases, while loss of function of any of the other subunits mostly causes a cancer phenotype [4]. This is the case even though all subunits are necessary for and contribute to the efficiency of the complex as a whole. In this work, we studied the outcomes of knocking down the expression of three of the four Sdh subunits in the established mitochondrial disease model, *Dictyostelium discoideum*. Our results showed that in *D. discoideum* a reduction in SdhA, B or C expression resulted in decreases in succinate dehydrogenase activity as in mammalian cells, although this reduction did not reach significance in our SdhB antisense-inhibited transformants.

It has been shown that SdhA activity assayed in vitro is dependent on its binding to the other subunits and mature free SdhA unbound to other subunits has very little catalytic activity [24]. In our semiquantitative western blots and quantitative in vitro activity assays, the subunit levels and associated Sdh activities were significantly reduced in both the SdhA and SdhC antisense strains. The lack of a significant decrease of Sdh activity in the SdhB transformants may be because the reduction of SdhB expression was not low enough. Unfortunately, this could not be verified because we did not have an antibody suitable for measuring SdhB protein expression levels. However, the reduced SdhB mRNA levels in these knockdown strains were accompanied by phenotypic abnormalities, the simplest explanation of which is that they were caused by reduced expression of the protein itself.

Despite the demonstrably lower Sdh enzyme activities in both the SdhA and SdhC knockdowns (Figure 1), Seahorse respirometry detected reduced Complex II activity in intact cells only in the latter (Figure 6E). This may be due to the difference in how Complex II activity is measured in the two methods. The in vitro Sdh activity assay measures, in crude cell lysates, the donation of electrons from experimentally provided substrate succinate, via FADH and the iron-sulphur cluster of SdhB to a colour-forming ubiquinone analogue (via Complex III/IV or AOX). This measure is thus independent of the cellular context, including the activity of the TCA cycle. In the Seahorse assay, the contribution of Complex II to maximum, uncoupled O_2_ consumption is measured in intact cells provided with excess TCA cycle substrates in the form of pyruvate and malate. The Seahorse assay may thus be more physiologically relevant, depending as it does on the cellular context, which determines the rate of provision of succinate to complex II by the TCA cycle and is subject to multiple layers of regulation, both at the protein expression and metabolic activity levels.

Our Seahorse results suggest a model for the consequences of SdhA knockdown in intact cells, according to which the reduced availability of the SdhA subunit is not the rate-limiting factor for maximal complex II activity. Instead, it rate limits and diminishes the flux of carbon through the TCA cycle, reducing both the rate of supply of TCA cycle intermediates for biosynthetic reactions and of electrons for biosynthesis and oxidative phosphorylation. The result is a new steady-state in which the lower ATP synthesis rates are accompanied by lower ATP steady-state levels (Figure 5E) and chronic activation of AMPK which homeostatically reduces cellular ATP demand by inhibiting biosynthetic processes including cell growth (Figure 5B) and mitochondrial biogenesis (Figure 5F). The mitochondrial membrane potential is higher (Figure 5G) because its use by complex V to drive ATP synthesis is reduced.

Chronic activation of AMPK has been reported in many pathological conditions such as polycystic kidney disease [25], Alzheimer’s disease [26], ischemia and aging [27,28]. One of the phenotypic consequences of chronic AMPK hyperactivity in the *Dictyostelium* mitochondrial disease model is the greater intracellular proliferation of the intracellular bacterial pathogen *Legionella pneumophila* [22]. We showed here that this is also the case in the SdhA knockdowns (Figure 5C), supporting the view that chronically hyperactive AMPK is responsible for many of the phenotypic outcomes of SdhA knockdown. If this is so, then AMPK knockdown in the same cells should reverse many of these outcomes. Our results here showed that when AMPK and SdhA were both knocked down in the same cells, the phenotypic consequences of SdhA knockdown were all reversed. In fact, in some cases (impaired phagocytosis and mitochondrial respiratory function) the reversal actually surpassed what was needed to restore wild type levels.

A traditional advantage of working with clonally grown model organisms, such as *Dictyostelium discoideum* in this work, is that independent, clonal mutants altered in a single target gene can be readily created and studied. This allows clear cause-effect relationships to be established experimentally without the unavoidable complications in clinical observational studies of multiple, unknown genetic modifiers elsewhere in the genome. On the other hand, those genetic modifiers elsewhere may play important roles in the penetrance and disease outcomes in humans and such effects would not be apparent unless explicitly tested. In *Dictyostelium*, it is easy to explicitly test the effect of potential modifiers as we have done here in the case of AMPK. Our results suggest that differences in AMPK expression or activity could be one such modifier in SdhA-deficient cells, with the phenotype being ameliorated by reduced AMPK activity or exacerbated by higher AMPK activity.

Not only were the phenotypic consequences of SdhA knockdown reversed by AMPK antisense inhibition, but the activity of SdhA itself was restored to normal. Since AMPK is known to activate the transcription of nuclear-encoded mitochondrial proteins, this result was unexpected. The simplest explanation is that the lower levels of SdhA mRNA in the knockdowns are translated at higher rates when AMPK is also knocked down. The canonical mechanism by which AMPK inhibits translation is by inhibiting TORC1, a known translational activator of mitochondrial biogenesis, including the proteins involved in oxidative phosphorylation. It would be valuable in future work to test this hypothesis that AMPK-mediated inhibition of TORC1 is responsible for many of the phenotypic outcomes of reduced SdhA activity.

Unlike in other *D. discoideum* cells with mitochondrial dysfunction, SdhA antisense-inhibited transformants exhibited decreased phagocytosis and no significant defect in plaque expansion rates on bacterial lawns. The phagocytosis defect was mediated by AMPK, as evidenced by the fact that it was reversed by AMPK knockdown. This finding suggests that under some circumstances AMPK hyperactivity can inhibit phagocytosis. What might those circumstances be? Recent data from our laboratory has shown that in oxidatively stressed cells, phagocytosis becomes sensitive to and inhibited by AMPK signaling [29]. Whilst ROS levels were slightly elevated in our Sdh knockdowns, they did not reach statistically significant levels (Figure 4D). However, a significant increase in the mitochondrial membrane potential was observed and it has been established by others that an increased MMP results in increased ROS production [30]. Reduced Complex II activity can also lead to increased ROS production [31,32].

As discussed above, the cytopathological consequences of SdhA knockdown can, in principle, all be traced to reduced supply of electrons both for biosynthesis and oxidative phosphorylation by the TCA cycle. Why would these same consequences not be apparent when the supply of other Sdh subunits is reduced? Our results do not provide an explanation, but they do confirm that in the *Dictyostelium* model the cytopathological consequences of reducing or losing SdhB or C function are unlike those caused by loss of SdhA. Unlike knocking down SdhA, knocking down either SdhB or SdhC resulted in defects in growth on bacterial lawns without any corresponding impairment of phagocytosis. This was unaccompanied by any impairment of mitochondrial biogenesis, ATP synthesis rates or ATP steady state levels. The SdhC knockdowns exhibited reductions in the mitochondrial components of basal respiration (statistically significant only in the case of the proton leak) and the mitochondrial components of the maximum respiratory capacity (significant only in the case of complex II activity). This was accompanied by elevated rates of nonmitochondrial O_2_ consumption, which is driven by cellular oxygenases that directly use molecular O_2_. These results suggest a dysregulation of cellular metabolism that is distinct from that caused by SdhA knockdown. On the other hand, the SdhB knockdowns exhibited an increase in respiratory function that reached statistical significance only in the case of the basal respiration rate, the maximum respiratory capacity and its major component, complex I activity.

Whatever the explanation of these distinct patterns of cytopathological outcomes is, they suggest that the catalytic subunit, SdhA, and its heterodimeric partner SdhB, may exist in the cell in more than one pool, perhaps in association with other proteins or alternative acceptors of the electrons from succinate. This would help explain differences in the phenotypic consequences of knocking down the different Sdh subunits. By way of a speculative example, if the SdhC/D heterodimer in the inner mitochondrial membrane is the preferred assembly partner for SdhA/B, then reducing its availability (eg., by knocking down or mutating SdhC) could cause accumulation of SdhA and/or SdhB in an alternative pool. Knocking down or mutating SdhA activity on the other hand would preferentially reduce its levels in the alternative, non-complex II pool. Another key TCA cycle enzyme, fumarate hydratase, whose mutation can cause cancer or a neurological phenotype, is known to be present in two pools, one in the mitochondria and one in the cytosol (Schmidt et al., 2020). A study by Raimundo et al. (2008) showed that a patient with a fumarase mutation and presentation of cancer had normal levels of mitochondrial fumarase but no cytosolic fumarase, suggesting different roles of the differentially localised fumarase.

The possibility of alternative pools and binding partners for SdhA has been little investigated. However, it has recently been suggested that when energy is low or when SdhB expression is reduced, an alternative assembly of Complex II is formed so that SdhA forms a complex with its two accessory proteins and is not bound by other subunits [33]. The authors termed this alternative assembly CII_low_ and noted that when SdhB was knocked out, the cells differentially regulated a number of proteins including upregulation of catabolic and salvage pathways. In these cells, this resulted in a decrease in mitochondrial respiration [33] rather than the increase we observed in this work. In our case, we are using a knockdown rather than a knockout strain and a simpler organism, but the premise remains that depletion of SdhB could result in the activation of compensatory pathways to restore the energy and redox imbalance. In *D. discoideum* this may result in the upregulation of these pathways so effectively in the SdhB case that respiration is actually increased beyond wild type levels.

In conclusion, our results reveal differences in the cytopathological outcomes of knocking down different Sdh subunits in *Dictyostelium*. Partial loss of SdhA resulted in mitochondrial dysfunction and AMPK-mediated phenotypic outcomes similar to those in previously studied *D. discoideum* mitochondrial disease models. Partial loss of SdhB or SdhC however resulted in defects in growth on bacterial lawns and unique effects on mitochondrial respiration.

## 4. Materials and Methods

### 4.1. Dicytostelium Discoideum Strains, Culture Conditions and Multicellular Development

All experiments were conducted using *D. discoideum* parental strain AX2 and transformants derived from it. For bacterial growth, the cultures were grown on lawns of *Enterobacter aerogenes* on SM agar (Formedium, Hunstanton, Norfolk, UK) at 21 °C as previously described [34]. Cells were also grown axenically in HL-5 (Formedium, Hunstanton, Norfolk, UK) liquid medium with shaking (150 rpm) at 21 °C. As a selective marker, G418 (ThermoFisher Scientific, Waltham, MA, USA) at 25 µg/mL was added to the growth media during subculturing for all the transformants. However, for all the phenotypic investigations, all antibiotics were excluded from the growth medium in order to exclude any possible antibiotic-associated effects. The transformants contained the constructs described below for antisense inhibition of expression of SdhA (pPROF227), SdhB (pPROF765) or SdhC (pPROF756).

The fruiting body morphology of AX2 and transformants was examined after growth on KK2 (20 mM potassium phosphate buffer, pH 6.4, 1.2% agar) agar plates for several days at 21 °C. Aerial view images were taken of the fruiting bodies using an Olympus S761TM dissecting microscope equipped with a Mitocam 2300TM camera.

### 4.2. Plasmid Construction

For the construction of the Sdh antisense plasmids, the following gene-specific primers were used to amplify a fragment of the genes using AX2 genomic DNA as a template. For the sdhA gene, a 1493 bp fragment was PCR-amplified using the primers 5′sdha2 (5′-GTCGCAGCACAAGGTGCTATTAATAATGC-3′) and 3′sdha1 (5′-GCGAATTCGGATCCATGAGCACCACGACTTTCTTT-3′). For the sdhB gene, a fragment 338 bp in size was PCR amplified using the primers SdhBF (5′-GCGAATTCGGTACCATTACCACACATGCAT-3′) and SdhBR (5′-GCGAATTCGGATCCGATCTTCAGAGAGGAT-3′). For the sdhC, gene a 225 bp fragment was amplified via PCR using the primers SdhCAS_F 5′-GCGAATTCCTCGAGGCACCACTTGTCAGAAATTG-3′), and SdhCAS_R (5′-GCGAATTCGGATCCCATAACGGCTGGTAATGGG-3′). The PCR products were subcloned into the EcoRI site of the pDNeo2 plasmid in the antisense orientation to create the constructs named pPROF227 (SdhA), pPROF765 (SdhB) and pPROF756 (SdhC). All the constructs created for this study were verified by sequence analysis at the AGRF. We did not deploy empty vector controls in this work, because it is well established that there are no adverse phenotypic outcomes in *Dictyostelium* of the vectors themselves, or vectors with antisense fragments cloned in the sense orientation, out of frame, and following a stop codon, or vectors expressing nonfunctional portions of *Dictyostelium* proteins or vectors ectopically expressing marker proteins such as GFP or aequorin that do not interact with other proteins in the cell (Kotsifas et al. 2002; Bokko et al., 2007; Annesley et al., 2007; Nebl and Fisher, 200; Ahmed and Fisher, 2006).

### 4.3. D. discoideum Transformation

AX2 cells were transformed with 15 µg DNA of each of the necessary constructs using the calcium phosphate DNA precipitation method [35]. All transformants were selected from isolated colonies on *Micrococcus luteus* lawns grown on SM agar supplemented with 25 µg/mL G418 [36] and subsequently subcultured and maintained on *E. aerogenes* lawns and axenically in HL-5.

### 4.4. Measurement of Construct Copy Number

To measure the number of copies of the antisense inhibition constructs per genome in the transformants, quantitative PCR (qPCR) was employed using a CFX Connect real-time PCR detection system and the iQ SYBR Green Supermix (Bio-Rad, Hercules, CA, USA) according to the manufacturer’s instructions. Firstly, genomic DNA was extracted from *D. discoideum* WT and transformants using DNAzol (Molecular Research Center, CI, Ohio), according to the manufacturer’s instructions and the resulting DNA was resuspended in 50 µL milliQ H_2_O containing 100 µg/mL RNaseI. The following primer sequences were used for amplification of a portion of each gene including *abpC* (encoding filamin), a single copy gene which was used as a loading control:

Filamin: Forward primer 5′-CCACAGAGATATTGGAGTTGCGTACC-3′, Reverse primer 5′-CAACTCAACCAATGTGCCTGCCAA-3′.

SdhA: Forward primer 5′-TGCTCGTGGTGAAGGTGGTTATCT-3′, Reverse primer 5′- ACATCACGAGAGGCTAAATCGGCT-3′.

SdhB: Forward primer 5′-CAACACAACCAACACCCCATT-3′ Reverse primer 5′-GTGAGGTTGGTTGGTATGGAG-3′

SdhC: Forward primer 5′-CTGGTCTTGCAGGTGTTACT-3′, Reverse primer 5′-GGGTATTGAGTATGGAGGAGTTG-3′.

Two standard curves were prepared. One was used for estimation of the total DNA loaded and deployed known quantities of pure genomic DNA of the parental strain AX2 with the filamin primers. The second was for estimation of the quantity of the plasmid construct in question and deployed purified plasmid DNA and the cognate gene primers of interest. The standard curves were used in combination with the known sizes of the amplified fragment and of the *D. discoideum* genome to estimate the construct copy number. These raw copy number estimates were then normalised against the estimated number of copies of the target gene in the control parental strain AX2 included in the same experiment as an internal control (bringing the control copy number to the known value of 1).

### 4.5. Calculation of mRNA Expression

Semiquantitative real-time RT-PCR was used to measure relative expression levels of SdhA, SdhB and SdhC mRNA. Real-time PCR was performed as described previously [37] using SensiFast SYBR and Fluorescein One-Step Kit and CFX Connect (Bio-Rad, Hercules, California, USA) real-time PCR detection system. Filamin was used as a loading control to estimate the total RNA loaded. The same filamin and Sdh primers were used as described in the previous section. RNA from WT and transformants was prepared using TriZol (Invitrogen, Carlsbad, CA, USA) according to the manufacturer’s instructions.

### 4.6. Western Blotting

Whole-cell extracts were prepared by lysing 5 × 10^6^ cells in 50 µL 2× Laemmli sample buffer (4% SDS, 20% glycerol, 0.004% bromophenol blue and 0.125 M Tris-Cl, pH 6.8). A 15 µL aliquot of cell lysate was heated at 95 °C for 10 min and then loaded onto 10% stain-free polyacrylamide gels for SDS-PAGE using a Bio-Rad MiniProtean II apparatus. The proteins thus separated were transferred to a PVDF membrane (Bio-Rad, Hercules, CA, USA) with the help of the Bio-Rad Mini Trans-Blot electrophoretic transfer cell. The membrane was probed with rabbit polyclonal anti-SdhA (1:1000, custom-made), and anti-SdhC (1:1000, Santa Cruz Biotechnology, Dallas, TX, USA) diluted in blocking buffer (1% casein, in TBST) overnight at 4 °C, followed by incubation in secondary goat anti-rabbit, HRP-conjugated (ThermoFisher Scientific, Waltham, MA, USA) for 1 h at RT. The protein bands of interest were visualized using Clarity ECL Western blotting substrate (Bio-Rad, Hercules, CA, USA) and acquired on an Amersham Imager 600 (GE Healthcare, Chicago, IL, USA) or a Chemidoc Touch (Bio-Rad, Hercules, CA, USA).

### 4.7. Succinate Dehydrogenase Activity Assay

Succinate dehydrogenase activity was determined following the reduction of the dye DCPIP (2,6-Dichlorophenolindophenol sodium salt hydrate) (an artificial electron acceptor) and succinate at 600 nm at 25 °C as described in [38]. Exponentially grown *D. discoideum* vegetative cells were harvested, washed, and resuspended in H_2_O to a density of 1 × 10^6^ /mL. The cells were lysed by quick freezing at −80 °C for 15–30 min. In a clear 96 well, flat bottom plate, 40 μL of succinate reductase assay buffer (10 mM KH_2_PO_4_, 2 mM EDTA, 1 mg/mL BSA, 80 μM DCPIP, 4 μM rotenone, 0.2 mM ATP, 10 mM succinate) was added, followed by 50 μL of cell lysate. The reaction was started by the injection of 10 μL of 80 μM decylubiquinone, while measuring the absorbance at 600 nm for 10–15 min using a Clariostar microplate reader and finalized by the complete inhibition of Complex II with the injection of 10 mM malonate.

### 4.8. Growth in Axenic Medium

*D. discoideum* AX2 and transformant strains were grown exponentially in HL-5 medium without antibiotics and then subcultured into fresh 50 mL HL-5 at a density of 1–2 × 10^4^ cells/mL and incubated shaking at 150 rpm at 21 °C. The cell densities were determined using a haemocytometer at 6–12 h intervals over 5 days and the generation times were estimated by log-linear regression during the exponential phase of growth using the R environment for statistical computing and graphics (http://www.R-project.org, accessed on 27 September 2021).

### 4.9. Growth on Bacterial Lawns

A scraping of amoebae from the edge of AX2 and transformant *D. discoideum* plaques was inoculated in the centre of an *E. coli* B2 lawn grown on Normal agar (20 g/L agar (ThermoFisher Scientific, Waltham, MA, USA), 1 g/L bactopeptone (ThermoFisher Scientific, Waltham, MA, USA), 1.1 g/L glucose, 1.9972 g/L KH_2_PO_4_, 0.356 g/L Na_2_HPO_4_*2H_2_O). The plates were incubated at 21 °C and the diameter of each plaque was recorded at intervals of 8–12 h for a period of 5–7 days. The results were analysed by linear regression using the R software to calculate the growth rate for each strain.

### 4.10. Endocytosis Assays

Phagocytosis by *D. discoideum* strains was measured by determining uptake of an *E.coli* strain expressing the fluorescent protein DsRed [39] as previously described [34]. Macropinocytosis rates were determined via measuring uptake of fluorescein isothiocyanate (FITC)-dextran (Sigma-Aldrich, average mol. Mass 70 kDa, St. Louis, MO, USA) as previously described [34].

### 4.11. Phototaxis Assay

Qualitative phototaxis was done as previously described [40]. Scrapings of amoebae from the edges of *D. discoideum* plaques grown on SM agar plates with a lawn of *E. aerogenes* were placed on the centre of charcoal agar plates (5% activated charcoal, 1.5% agar). The plates were incubated at 21 °C for 2 days with a single lateral source to allow slugs to form and migrate. The slug trails were examined, transferred to PVC discs and stained with Coomassie Blue and then photographed for further analysis.

### 4.12. Seahorse Respirometry

A Seahorse Extracellular Flux Analyser (Agilent Technologies, Santa Clara, California) was used for mitochondrial respiration assays as described previously [41]. *D. discoideum* AX2 and transformed cells were grown axenically in HL-5 medium to exponential phase, washed and resuspended in SIH (Formedium, Hunstanton, Norfolk, UK) media supplemented with 20 mM pyruvate and 5 mM malate, pH7.4. Each strain was inoculated onto 8 wells for transformants or 4 wells for AX2 of a 24-well cell culture plate, pre-treated with Matrigel, to ensure cell attachment during the assay. Basal Oxygen Consumption Rates (OCR) were measured, followed by the injection of a series of mitochondrial inhibitors and reagents: [10 μM DCCD (N,N0-dicyclohexylcarbodimide, an ATP synthase inhibitor (Sigma-Aldrich, St. Louis, MI, USA), 10 μM CCCP (carbonyl cyanide 3-chlorophenol hydrazone, a protonophore (Sigma-Aldrich, St. Louis, MI, USA)], 20 μM rotenone [Complex I inhibitor (Sigma-Aldrich, St. Louis, MI, USA)] and either 10 μM antimycin A [Complex III inhibitor (Sigma-Aldrich, St. Louis, MI, USA)] or 1.5 mM BHAM [benzohydroxamic acid, alternative oxidase (AOX) inhibitor (Sigma-Aldrich, St. Louis, MI, USA)]. Measurement cycles consisting of 3 min of mixing, 2 min wait, and 3 min measurement time were completed before and after each sequentially added drug, at least two cycles per condition. The measurements could then be used to calculate the contribution of different complexes and components to mitochondrial respiration.

### 4.13. Mitochondrial Mass and Mitochondrial Membrane Potential

Measurements of mitochondrial mass and mitochondrial membrane potential were performed simultaneously using the mitochondrial stains Mitotracker Red CMXRos and Mitotracker Green FM (ThermoFisher Scientific, Waltham, MA, USA). *D. discoideum* AX2 and transformed cells were harvested, resuspended in LoFlo HL5 to a density of 1 × 10^6^ cells/mL and 100 μL of each strain was seeded into 6 wells of a 96-well black plate and allowed to equilibrate for 40 min. The mitochondrial stains (Mitotracker Red 200 nM and Mitotracker green, 400 nM) diluted in LoFlo HL5 medium were added to 2 wells each and further incubated for 1 h. For each strain, duplicate wells of unstained cells were used as blank. The fluorescence measurements were performed using a Clariostar microplate reader (BMG) with excitation and emission set at 470 nm 515 nm respectively for Mitotracker Green and 570 nm and 620 nm for Mitotracker Red. The mitochondrial mass was calculated by subtracting the fluorescence of the unstained well from that of the Mitotracker Green-stained wells, while the mitochondrial membrane potential was the background-subtracted Mitotracker Red fluorescence divided by the background-subtracted Mitotracker Green fluorescence. The parental AX2 strain was used for assay normalization.

### 4.14. ATP Steady-State Levels

The ATP determination kit (ThermoFisher Scientific, Waltham, MA, USA) was used to determine the ATP content for the *D. discoideum* AX2 and transformant strains according to the manufacturer’s instructions. *D. discoideum* strains were grown to exponential phase 1–3 × 10^6^ /mL, harvested by centrifugation and 1 × 10^5^ cells were aliquoted into duplicate sterile Eppendorf tubes. Cells were lysed and ATP was extracted by the addition of 100 µL 1% (*w*/*v*) TCA. The TCA was neutralized by the addition of 900 µL of 28 mM tricine buffer pH 8.25 and 5 µL of supernatant was transferred to a fresh microcentrifuge tube and 45 µL of reconstituted luciferin reaction buffer was added and immediately measured. The intensity of the light emitted which is proportional to the amount of ATP was measured in a Modulus Fluorometer (Turner Biosystems, Sunnyvale, CA, USA) using the luminometer module. A separate calibration curve relating the luminescence signal with known amounts of ATP was used to estimate the total amount of ATP in each sample.

### 4.15. Reactive Oxygen Species (ROS) Assay

ROS measurements were performed using DCFDA (2′,7′-dichlorofluorescein diacetate, Sigma-Aldrich, St. Louis, MI, USA), a fluorogenic, cell-permeant reagent that is able to detect hydroxyl, peroxyl and other reactive oxygen species within cells. Once DFCDA diffuses into the cells, it is deacetylated by cellular esterases and then oxidized by the intracellular ROS into the green fluorescent DCF. *D. discoideum* AX2 and transformed strains grown axenically were harvested by centrifugation at 1000× *g* for 1 min, resuspended in LoFlo HL5 at a density of 1 × 10^6^ /mL and 100 μL of the cell suspension was plated into 4 wells of a 96 well black, clear flat bottom plate (Corning Inc., Corning, NY, USA). After one hour of incubation at RT, 100 μL of DCFDA was added to two wells to a final concentration of 20 μM, and two wells were used with no reaction mix for background subtraction. The fluorescence was measured using a Clariostar plate reader at 485 nm excitation, 535 nm emission. The background values were subtracted from the stained values and then averaged and normalized to values from parental AX2 strain.

### 4.16. Legionella Proliferation Assay

The *Legionella* proliferation assay was performed as described previously [22]. Axenically grown cells were harvested by centrifugation at 1000× *g*/3 min, washed twice with Sorensen 1×C buffer (17 mM KH_2_PO_4_/Na_2_PO_4_, 50 mM CaCl_2_, pH 6.0) and the pellet was resuspended in MB medium (0.7% yeast extract, 1.4% proteose peptone, 0.062% Na_2_HPO_4_·2H_2_O, 0.049% KH_2_PO_4_, pH 6.9) at a density of 5 × 10^5^ cells/mL. For each strain, 1 × 10^5^ cells were transferred into 6 wells of a 96-well tissue culture plate (Corning Inc., Corning, NY, USA) and allowed to adhere for 30 min. The *Legionella pneumophila* Corby strain was grown on Buffered Charcoal Yeast Extract Agar (BCYE agar) supplemented with chloramphenicol 5 μg/mL at 37 °C with 5% CO_2_ for 3 days. The bacteria were harvested and resuspended in distilled water, diluted in MB to a density of 1 × 10^6^ bacteria/mL and used to infect *D. discodeium* in each well at an MOI of 1:1. This was achieved by reading the OD of the bacterial suspension, assuming that an OD_600_ of 1 equates to 10^9^ bacteria/mL. To ensure initial adherence the plate was centrifuged at 700× *g*/10 min. At each time point (0, 2, 24, 48, 72 and 96 h) 50 µg/mL of gentamycin was added to each of the wells and incubated for 30 more min to achieve the lysis of any uningested bacterial cells. The *D. discoideum* amoebae were resuspended, transferred to 1.5 mL microfuge tubes and pelleted by centrifugation at 13,000× *g* for 10 min. The cells were washed twice in ice-cold Sorensen’s 1 ×C buffer and resuspended in 300 µL Sorensen’s 1×C buffer, supplemented with 0.02% saponin and lysed by vigorous vortexing for 10 sec. A dilution series of the lysate from 10^−1^ to 10^−4^ was prepared and spread-plated on BCYE plates without chloramphenicol. The plates were incubated at 37 °C with 5% CO_2_ for 3–4 days prior to counting the number of colonies to calculate the number of viable *L. pneumophila* for each time point.

## Data Availability

Not applicable.
